# Parkinson’s disease-associated camptocormia: biopsy-confirmed focal myositis and treatment options

**DOI:** 10.1007/s00415-025-13468-4

**Published:** 2025-10-23

**Authors:** Angela Rosenbohm, Nam Nguyen-Younossi, Jan Kassubek

**Affiliations:** Department of Neurology, University Hospital Ulm, Oberer Eselsberg 45, 89081 Ulm, Germany

**Keywords:** Parkinson’s disease, Camptocormia, Myositis, Immunosuppression

## Abstract

**Background:**

The etiology of the disabling symptom camptocormia/dropped head in Parkinson’s disease (PD) is still debated. Many PD patients develop involuntary flexion of the spine in an upright position that does not respond to dopaminergic treatment.

**Objectives:**

To study the histopathology of paraspinal muscles in PD-associated camptocormia with potential consequences for therapy.

**Methods:**

We report a cohort of 66 patients with PD (mean age 68.4 ± 8.1 years) and associated camptocormia according to consensus criteria. Paravertebral muscle biopsy was performed in all patients with consecutive histopathological analysis.

**Results:**

Forty-two patients (64%) showed inflammatory myositis in the paravertebral muscles with features of T-cell dominant myositis with additional necrosis, in addition to moderate myopathic changes. Thirty-four of these 42 patients with myositis received immunosuppression according to general clinical guidelines for myositis (i.e., prednisolone, often followed by azathioprine) in addition to PD medication. Twenty-three/34 patients (68%) on prednisolone showed a partial or complete improvement of the muscle weakness. This effect persisted after switching from prednisolone to the long-term immunosuppressant. Three/34 patients were lost to follow-up, and 8/34 did not improve.

**Conclusions:**

In this cohort of PD patients with camptocormia, muscle biopsy demonstrated local myositis in addition to myopathic changes in 64% of the cases. About two-thirds of these patients benefited from prednisolone therapy, as a symptomatic therapeutic option for muscle weakness. We recommend a histological work-up of the clinical diagnosis PD-associated camptocormia given that effective therapeutic options are available.

## Introduction

The ‘classical’ presentation of camptocormia is characterized by 45° or more forward flexion of the cervical or thoracolumbar spine in upright position caused by a variety of neurological and musculoskeletal disorders [1] and has been initially described more than 200 years ago [2]. In 2018, consensus was reached within an expert group that camptocormia is defined as “an involuntary flexion of the spine appearing during standing or walking and resolving in the supine position of at least 30° at the lumbar fulcrum (L1-sacrum, hip flexion, i.e., lower camptocormia) and/or at least 45° at the thoracic fulcrum (C7 to T12-L1, i.e., upper camptocormia)” [3], and the associated condition of anterocollis/dropped head was defined as a forward flexion of the head and neck of at least 45° [4]. This “bent spine” or “camptocormia” might be caused by various neurological conditions, e.g. inflammatory muscle diseases or hereditary myopathies are known to be causes for paraspinal muscular weakness [5–11].

The occurrence in association with Parkinson´s disease (PD) or other neurodegenerative parkinsonism is recognized as a severe manifestation of postural abnormality and occurs with a frequency of < 6% [12], in some studies up to 18% [13] in advanced Hoehn and Yahr stages and 11.2% in a large study with 811 PD patients [14]. The overall prevalence of axial postural abnormalities in PD including Pisa syndrome was reported to be 22.1% in a recent review [1]. Most studies identified advanced disease stage and higher burden of motor symptoms as possible clinical predictors [15]. In PD, it is still debated whether camptocormia is due to the neurodegenerative disease process itself [16, 17], medication [18], muscle overuse in dystonic symptoms [19], proprioceptive dysregulation or an associated myopathy of unknown origin [13, 20]. Alternatively, these focal weaknesses of the paravertebral muscles were discussed to be a secondary focal myositis related to prolonged stooped posture [21].

Diagnostic work-up includes evaluation of axial posture, clinical assessment of medication, and may include MRI and electromyography. Correlates of altered paraspinal muscles in PD patients could also be observed in paraspinal MRI, which showed edema, fatty degeneration, and atrophy [22]. Dixon magnetic resonance technique also showed a higher fat fraction in paraspinal muscles compared to leg muscles in healthy subjects [23].

Muscle biopsy is not commonly included in the diagnostic work-up of postural abnormalities in PD [14, 24]. Reports on histopathological diagnosis in paravertebral muscles in PD are limited to case series scarcely exceeding 20 patients [22, 25–31]. Variable histological features were revealed in PD-associated camptocormia so far, i.e., myofibrillar disorganization, necrotizing myopathy, myositis, mitochondrial abnormalities, and also non-pathological biopsies or unspecific alterations [22, 25–27].

Based on a previous case series in PD-associated camptocormia in which muscle biopsies showed mitochondrial disease or myositis features [26], the present study aimed to analyze the association between the results of local muscle biopsy in paravertebral muscles and clinical therapeutic options. In non-PD-related paravertebral myositis, the treatment of inflammatory myopathies with immunotherapy often results in at least partial improvement in more than 50% of the patients and regression of camptocormia, especially in patients aged < 70 years [10].

## Methods

Skeletal muscle from 66 paravertebral muscles (multifidus *n* = 53, trapezius *n* = 11, sternocleidomastoideus *n* = 1, levator scapulae *n* = 1, splenius capitis *n* = 1) was obtained from PD patients for diagnostic reasons as part of the clinical work-up, including the routine procedure of muscle biopsy at this localization. This study was conducted according to the Helsinki Declaration. All included patients gave informed consent prior to study inclusion. The study was approved by the Ethics Committee of the University of Ulm (reference no. 20/10).

### Patients

The demographic and clinical data of the patient cohort are summarized in Table 1. A total of 66 patients (male: *n* = 42; mean age at biopsy 68.4 ± 8.1 years, range 49–81 years) are reported. All patients were diagnosed with PD (according to classical clinical UK brain bank criteria or current MDS criteria [32] after their publication, respectively) and presented with camptocormia according to consensus criteria [3]. The patients who were diagnosed with camptocormia before the publication of those criteria were re-assessed based on clinical charts. No patients were included who presented with Pisa syndrome. In accordance with previous clinical studies [14], all PD patients with camptocormia did not have any weakness in distal skeletal muscles of the limbs. All patients had been referred to the Department of Neurology, University Hospital Ulm, Germany for diagnostic procedures during the years 2003–2024. All patients included in the study received paravertebral muscle MRI, electromyography (contralaterally to biopsy site), and muscle biopsy for clinical diagnostic reasons. Equivalent dosing of levodopa was calculated according to Tomlinson et al. [33] and Jost et al. [34]. Thirteen patients of this cohort have been published previously [26] concerning the biopsy results.

**Table 1:** Demographic and clinical data of the patient cohort

PD patients (*N* = 66)	
Age at muscle biopsy (years), mean (SD)	68.4 (8.1)
Male sex, *N* (%)	42 (64)
Type of PD, tremor type, *n* (%)	5 (8)
Type of PD, bradykinetic/rigid type, *n* (%)	46 (70)
Type of PD, mixed type, *n* (%)	15 (23)
Modified H&Y stage, mean (SD)	3.0 (0.6)
UPDRS III score on state, mean (SD)	20.6 (5.5)
Duration of PD in years, median (25%; 75% percentile)	8 (4,25; 11)
Duration of CC in months, median (25%; 75% percentile)	12 (6; 30)
l-Dopa monotherapy, * n* (%)	5 (8)
Therapy naive PD, * n* (%)	1 (2)
Dopamine agonist (DA) monotherapy, *n* (%)	6 (9)
l-Dopa + DA, *n* (%)	54 (82)
Other antiparkinsonian drugs, *n* (%)	34 (52)
l-Dopa equivalent daily dose, mg, mean (SD)	862 (311)
Ongoing pharmacological PD therapy, *n* (%)	66 (100)
CK (IU/l), median (25%; 75% percentile)	133 (96; 252)
EMG myopathic, *N* (%)	35 (56)
MRI edema, *N* (%)	32 (58)
MRI atrophy, *N* (%)	9 (16)
MRI fatty degeneration, *N* (%)	22 (40)
MRI Contrast medium enhancement, *N* (%)	25 (45)
Biopsy: myopathy, *N* (%)	63 (95)
Biopsy: myositis or immune-mediated necrotizing myopathy, *N* (%)	42 (64)
Prednisolone therapy, *N*	34
Positive effect of prednisolone, *N*	23

### Electrophysiology

Electromyographical studies (EMG) were performed during the diagnostic work-up prior to muscle biopsy. EMG results were obtained from individually selected muscles according to clinical findings and patient history. The reports were evaluated for spontaneous activity, motor unit action potentials (MUAPs), and recruitment during maximum innervation. According to comparison with reference values [35], EMG results were classified as myopathic, neurogenic, or normal.

### Muscle MRI

Fifty-five patients underwent an MRI examination of the paravertebral muscles (field of view level according to each patient’s individual symptoms) using a 1.5 T MRI scanner (Magnetom Symphony, TIM, Siemens Healthcare, Erlangen, Germany). The routine MRI examination was conducted with the patient lying supine using a phased array body coil. All images were acquired in axial scan planes using fast-spin echo T1-weighted (TR/TE, 932/10; number of signal averages, 2; FOV: 439.1 × 330 mm), STIR (TR/TE, 7310/56; inversion time 140 ms; number of signal averages, 1; FOV: 439.1 × 330 mm), and T1-weighted fat saturated post contrast (TR/TE, 831/10; number of signal averages, 2; FOV: 439.1 × 330 mm) for all patients. For contrast medium application, 0.1–0.2 ml/kg of contrast medium (Bracco, Italy) was injected automatically at a rate of 1 ml/s, followed by 10 ml saline using an automatic injector (Medrad, Bayer, Germany).

### Muscle biopsy and histopathology

After written informed consent, a sample of approximately 100 mm^3^ from the muscle of the paravertebral muscles (mostly multifidus) was removed under local anesthesia. The sampling site varied vertically according to the kink region of the camptocormia and was located contralateral to the electromyography insertion site. The biopsies were snap-frozen for 2 min in isopentane immediately after their collection. After drying on dry ice for 10 min, the biopsies were stored at − 80 °C until the muscle sections were prepared.

Histopathological analysis of all muscle biopsies was performed according to standardized operational procedures [36–38]. Ten-micron frozen sections were stained with hematoxylin and eosin (HE), Gomori trichrome (Gomori), nicotinamide adenine dinucleotide dehydrogenase (NADH), cytochrome c oxidase/succinate dehydrogenase (COX/SDH), acid phosphatase, elastica-van Gieson (EVG), periodic-acid Schiff (PAS), adenosine triphosphatase (ATPase) at pH 4.3, 4.6, and 10.35, respectively, and oil-red O (ORO) [36]. Histopathological findings were categorized as myopathy, inflammation/myositis, mitochondriopathy, or neurogenic atrophy [37, 38].

Myopathic changes were qualitatively and quantitatively evaluated concerning shape and size, caliber variation of muscle fibers, position of nuclei, structural defects or changes in individual muscle fibers, acid phosphatase activity, distribution of connective and adipose tissue, fiber type distribution, splitting, presence of lymphocytic infiltration, vacuoles, glycogen, lipid droplets, and oxidative reactions as described in Dubovitz and Sewry [37]. When the biopsy indicated inflammatory changes, immunohistochemistry with antibodies against the epitopes of MHC1, CD4, CD8, CD20, and CD68 was complemented.

Myopathic changes were diagnosed when histopathology showed abnormal findings in a combination of increased fiber size variability with endomyseal fibrosis, fiber splitting, and whorled fibers, centralized nuclei, rounded fiber shape, and necrosis. Distinctive pathological changes, as well as an increased amount of adipose tissue, were also included in finding the final histopathological diagnosis, addressed as “myopathy” in this study.

To be regarded as “inflammatory,” histopathology indicated myopathic changes or normal muscle in combination with inflammatory lymphocyte infiltration as well as specific features like perifascicular atrophy, rimmed vacuoles, or MHC1 upregulation in muscle fibers other than single fiber atrophy. In this study, the term “myositis” was used for these diagnoses. Mitochondriopathy was determined as seeing ragged red fibers and/or COX-negative muscle fibers in the biopsy.

Neurogenic atrophy consisted of atrophic fibers of both types in reticular distribution or small/large groups of atrophic fibers of both fiber types without any myopathic changes.

All statistical analyses and visualizations were performed using Prism Version 10.6 (GraphPad, San Diego, CA, USA).

## Results

### Clinical and histopathological data

The mean age of the PD patients was 68.4 ± 8.2 years (range 49–81). Dopaminergic treatment consisted of levodopa in 56/66 patients and dopamine agonists in 62/66; 10 patients were treated with dopamine agonists alone, while the others received a combination with levodopa. Mean levodopa equivalent daily dose [33, 34] was 862 ± 311 mg. Creatine kinase was elevated in some patients (20/59 patients); the median creatine kinase value was 133 U/l (25th percentile 96; 75th percentile 252 U/l; the maximum was 1360 U/l).

Out of 62 patients, EMG revealed normal/myopathic/neurogenic muscle action potentials in 14/27/7, respectively, while 8 patients showed a mixture of neurogenic and myogenic potentials. In 6 patients, spontaneous activity was detected.

Out of 55 patients who received paravertebral MRI (53 with contrast agent), 32/55 showed muscle edema in the paravertebral multifidus muscles, 9/55 showed local paravertebral muscle atrophy, and 22/55 showed fatty degeneration of muscles. In addition, 25/53 demonstrated regional contrast enhancement of the paravertebral muscle. 23 patients out of 32 with MRI muscle edema had a histologically proven myositis (Table 1).

**Fig. 1:**
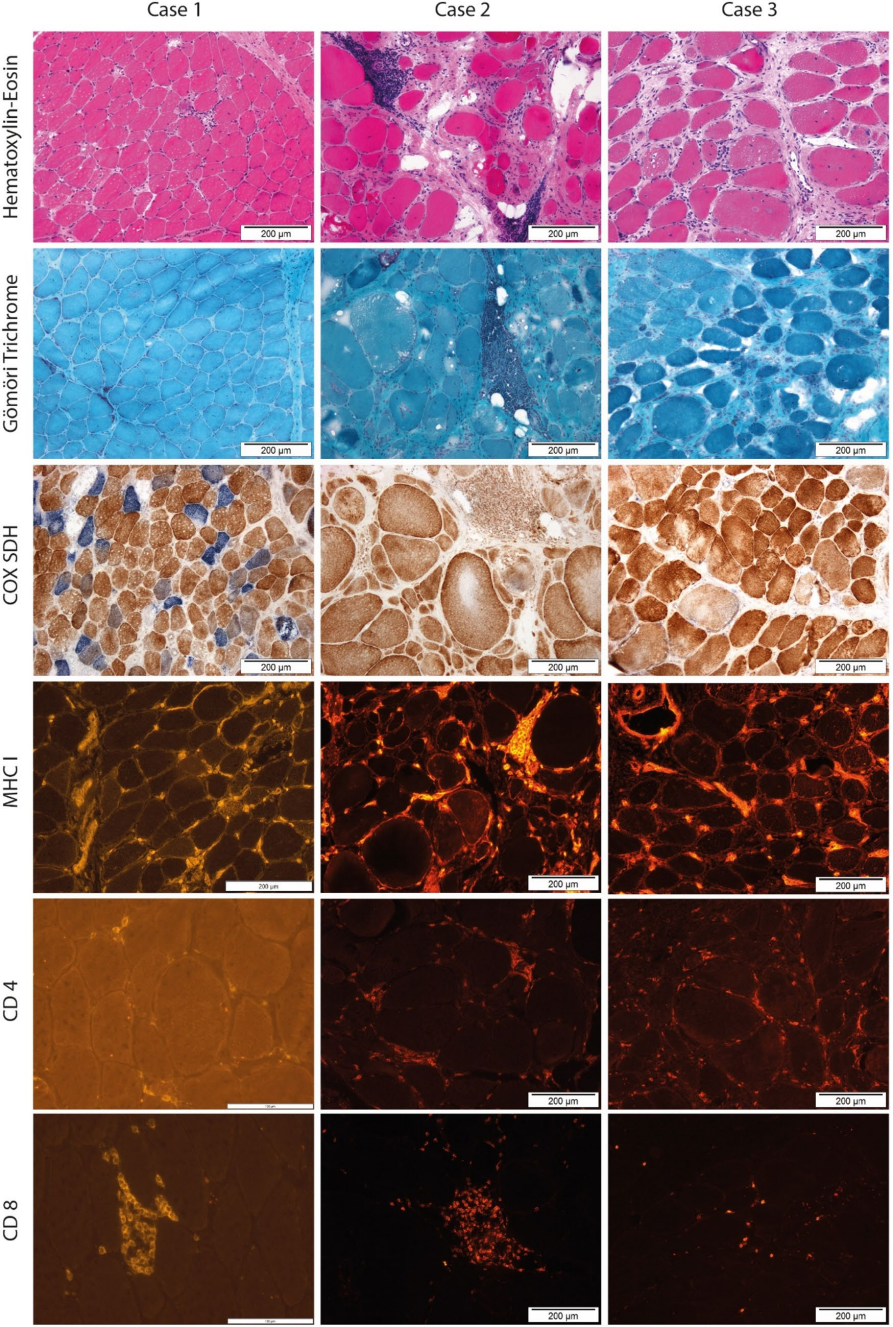
Histological characterization of three different biopsies with a broad spectrum of inflammatory abnormalities (each column representing one distinct biopsy). The extent of lymphocyte infiltrations, mitochondrial alterations with COX negative muscle fibers, and the amount of CD4+ or CD8+ T lymphocytes are shown in representative sectors of the biopsy

Muscle biopsy according to standard operating procedures was successfully performed without any complications in all patients. Histopathologic work-up showed myopathy in 63/66 paravertebral biopsies. More specifically, microscopic examination of muscle tissue (Table 2) showed inflammatory myopathy in 42/66 samples, specified as myositis in 38/66 biopsies, defined by perivascular and endomysial inflammatory cell infiltrates and a necrotizing immune myopathy in four patients, i.e., no inflammatory cells aside from myophagocytosis of necrotic fibers plus MHC1 upregulation. In the patients with myositis, CD68-positive macrophages and CD4-positive and/or CD8-positive T cells were observed more frequently than CD20-positive B cells. Diffuse or multifocal MHC-1 immunostaining of non-necrotic muscle fibers was evident in *n* = 28 (67%) of the patients with myositis or necrotizing myopathy. The distribution pattern of MHC-1 staining predominantly consisted of endomyseal clusters of MHC-1 positive myofibers, whereas perifascicular MHC1 staining pattern (like in dermatomyositis) was not observed in any of the cases. Representative histological staining samples are presented in Fig. 1.

**Table 2:** Muscle histopathology features in patients with PD and camptocormia

Paravertebral muscle biopsy, *N* = 66	*N*	Specification of inflammatory markers in IHC	
Inflammation (MHC1 expression and/or T-cell infiltration)	42/66	Perifascicular inflammation	0/42
		Endomyseal inflammation	38/42
		MHC1 expression	38/42
		CD4 T-cell infiltration	25/42
		CD8 T-cells surrounding or invading non-necrotic fibers	34/42
		CD68 + macrophages	33/42
Necrotic and/or regenerating fibers	51/66		
Myopathic features	63/66		
Splitting /whorled fibers	40/66		
Rounded fiber shape	62/66		
Endomyseal fibrosis	49/66		
Increased variability in fiber size	58/66		
Type 1 predominance	28/66		
Centralized nuclei	59/66		
Granulomatous lesions	0/66		

*IHC* immunohistochemistry, *MHC-1* major histocompatibility complex 1

**Fig. 2:**
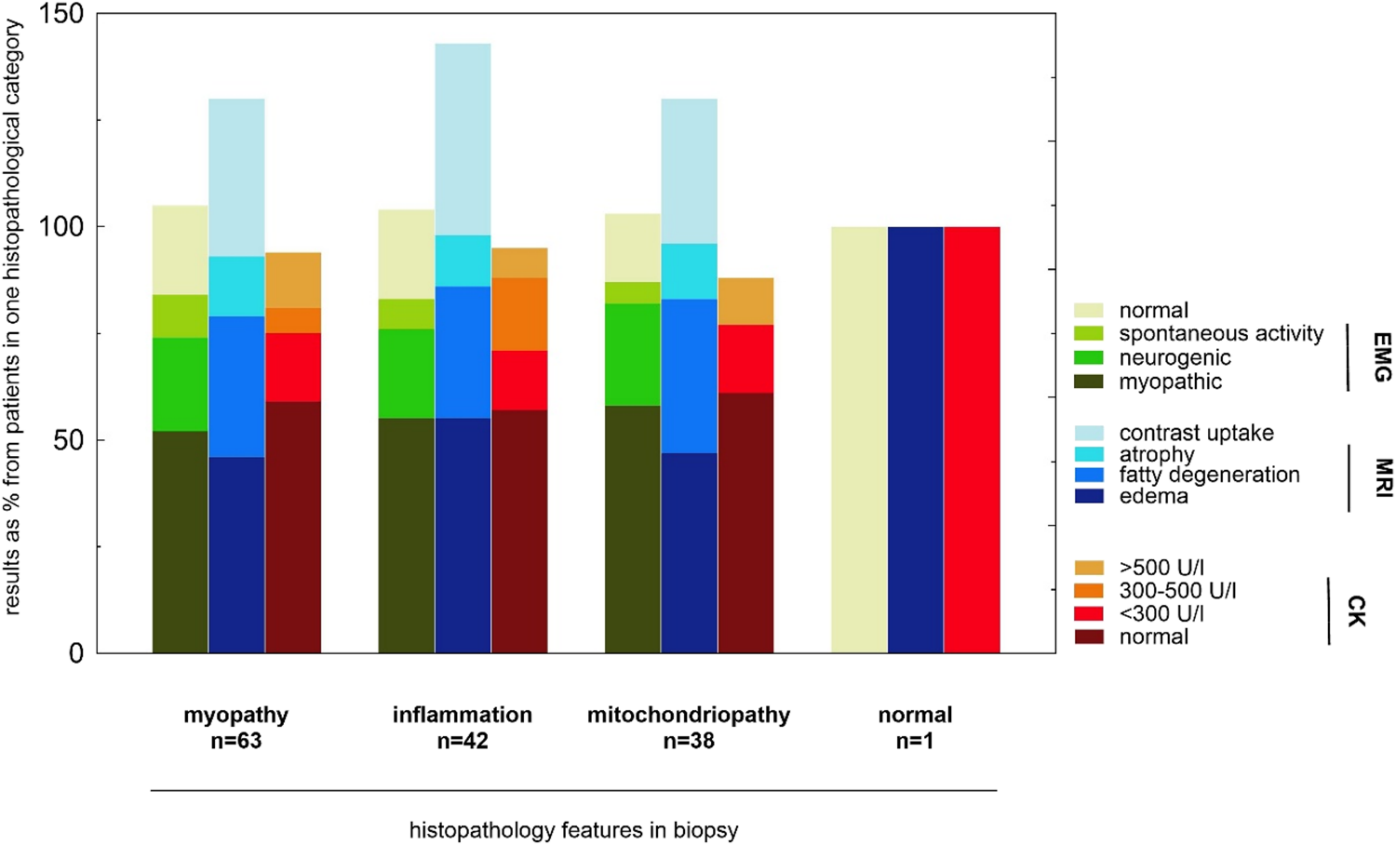
Diagnostic findings in EMG, MRI, and CK in % of the histopathological categories obtained in biopsy. Numbers are given as % of total counts as indicated in histopathology on the *X* axis; multiple entries were possible for EMG and MRI features in the case of combined occurrence. This may lead to a sum score of more than 100%. *MRI* magnetic resonance imaging, *EMG* electromyography, *CK* creatine kinase

Table 3 summarizes the MRI and EMG findings of the 66 patients with respect to the classification obtained by biopsy. Figure 2 presents the diagnostic findings in MRI and EMG and the creatine kinase values in the histopathological categories obtained in biopsy. Patients with inflammation in biopsy showed creatine kinase values of up to 500 U/l. EMG and MRI features could not distinguish between myositis and myopathy, showing almost equal percentages of MRI patterns in both groups (both myopathy and inflammation were accompanied by edema, fatty degeneration, atrophy, and contrast uptake in 46/55%, 33/31%, 14/12%, and 37/45%, respectively). Edema and contrast uptake were observed across all pathologies except for the one normal biopsy.

**Table 3:** MRI and EMG findings of *n* = 66 patients according to the biopsy features

	MRI (*n* = 55)				EMG (*n* = 62)			
	Edema	Fatty degen	Atrophy	CM	Normal	PSA	Myopathic	Neurogenic
Myopathy, *n* = 63	29	21	9	23	13	6	33	14
Inflammation, *n* = 42	23	13	5	18	9	3	23	9
Mitochondriopathy, *n* = 38	18	15	5	13	6	2	22	9
Normal, *n* = 1	1	0	0	0	1	0	0	0

MRI and EMG findings of *n* = 66 patients according to the biopsy features. Numbers are given as total counts

*MRI* magnetic resonance imaging, *EMG* electromyography, *fatty degen* fatty degeneration, *CM* contrast medium uptake, *PSA* pathological spontaneous activity

### Clinical treatment

**Fig. 3:**
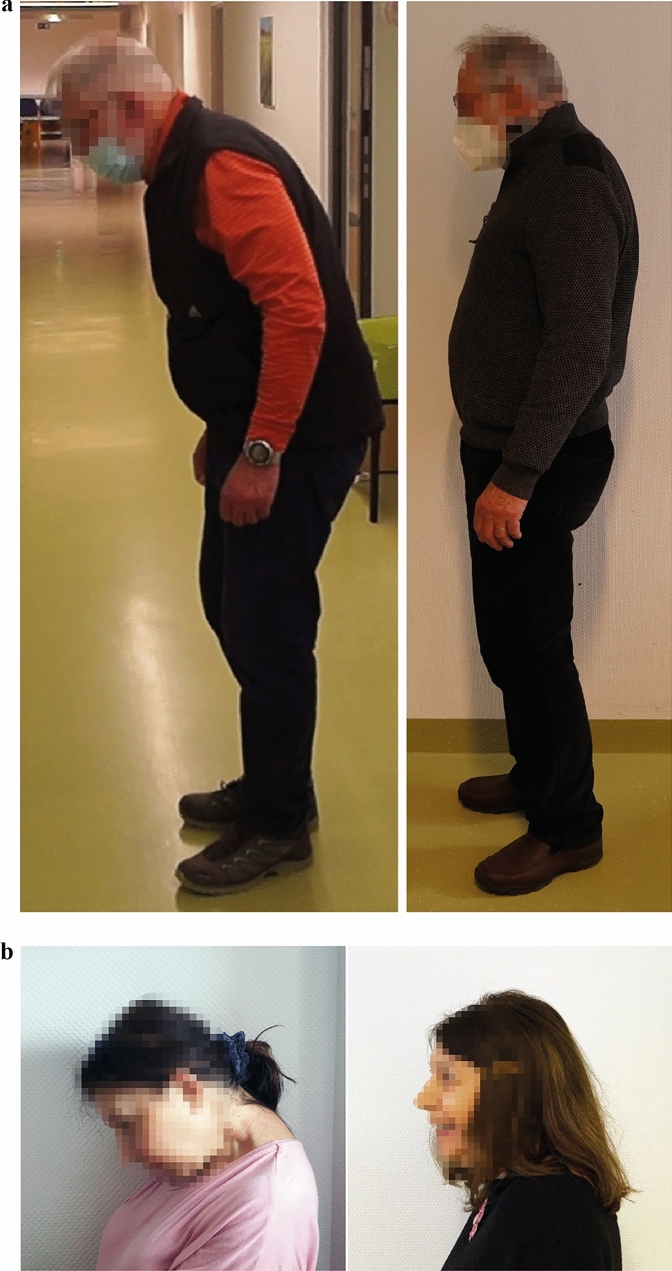
Clinical development of two myositis patients **A** after 6 months of prednisolone therapy and **B** after 1 year of azathioprine therapy

After histological work-up, 34/42 patients with inflammation received immunosuppressive therapy (prednisolone *n* = 34, azathioprine *n* = 7 after initial positive prednisolone effect), according to national guidelines for myositis, in addition to medication targeted at PD which was not modified while immunosuppressive therapy was started. Twenty-three/34 patients on prednisolone reacted with a partial or complete improvement of the postural abnormality (improvement of 1 grade in MRC scale and/or partial or complete normalization of the posture angle). Given the exploratory, non-standardized design, the improvement was defined as a reduction of the angle of involuntary flexion of the spine at the lumbar fulcrum or at the thoracic fulcrum or with respect to the forward flexion of the head/neck, respectively, depending on the individual patient’s clinical presentation. However, no quantitative assessment was performed, but the judgement encompassed global impression of the clinical improvement (by a movement disorders specialist together with a myopathies specialist, JK and AR) and the patient’s statement on reduction of clinical burden. This effect could also be sustained after switching from prednisolone to another individually chosen steroid-sparing long-term immunosuppressant; to this end, according to national guidelines for myositis, azathioprine was chosen and prednisolone tapered to a minimal dose for sustaining the positive effect on muscle weakness. Three/34 patients could not be followed up, 8/34 reacted with no improvement to prednisolone so that the medication was stopped. The individual duration of immunosuppressive therapy was between six months and several years, as supposed in the national myositis guidelines. One patient was tapered in azathioprine after 2 years due to spinalioma and relapsed for dropped head. Side effects were in the well-known spectrum of azathioprine therapy. Five patients who had no myositis in the biopsy were empirically treated at the individual level with prednisolone and showed no effect. Two typical patients and their corresponding clinical improvement after prednisolone therapy are shown in Fig. 3.

## Discussion

In this cohort of 66 PD patients with a clinical diagnosis of camptocormia, each patient received histopathological workup with muscle biopsy from the paraspinal muscles. The vast majority of 95% showed myopathic alterations in the muscle tissue, which were classified as inflammatory myopathy in 67% (myositis, *n* = 38; necrotizing immune myopathy *n* = 4). It has to be noted that these results do not differentiate if the (inflammatory) myopathy is the primary or a secondary process in the development of camptocormia, i.e., if it is a primary pathogenic mechanism or a secondary epiphenomenon due to chronic mechanical strain. Irrespective of the causality, histopathology identified local myopathy as a major contributor to the postural abnormality. Previous biopsy studies have not identified aberrant protein aggregation or signs of mitochondriopathy [25]. In PD, the pathophysiological concept of camptocormia is not established yet. Camptocormia in PD was interpreted as dystonia, or it was discussed that it may be due to a dysregulation of proprioception at the level of the central nervous system [18]. In the same line of the interpretation as a secondary clinical presentation, Geroin and colleagues argued that camptocormia might be a consequence of overusing paraspinal muscles due to rigidity in the context of underlying disease [24], but the authors did not specifically address the inflammatory component which was identified in our study. Discussions have to be considered in the light of reports of biopsies of healthy individuals’ paraspinal muscles, which showed more abnormalities compared to skeletal muscles in the arms or legs [25, 36], mimicking myopathic changes like increase in fiber size variability, type 1 hypertrophy, rounded fiber shape, and endomysial fibrosis as well as replacement by adipose tissue. Partial myopathic features and reduced oxidative enzyme activities, therefore, represent a normal variation at this location [36].

As a straightforward therapeutical consequence of the observed inflammatory muscle alterations, we were strictly relying on the histopathological diagnosis in analogy to clinical pathways of myositis as diagnosed by muscle biopsies of the limbs [39, 40] and recommended an individual therapy for those patients with histologically proven inflammatory myopathy. To this end, patients with inflammatory findings in biopsies were treated according to national and international standards for myositis with immunosuppressive agents like prednisolone or azathioprine (alternatively, mycophenolate-mofetil which was not used in our patients), as recommended in Vermaak et al. [40], and the clinical outcomes were assessed. To the best of our knowledge, this is the first large cohort of paravertebral myositis patients in PD, presenting with camptocormia, with a clinical follow-up regarding immunosuppressive treatment options. In the 34 patients with inflammatory myopathy, *n* = 23 (67%) showed a clinical benefit with improvement of the postural abnormality, which is remarkable given the limited treatment options in the literature in this severely disabling condition [15].

Myositis in other regions of the body is a treatable disease with an excellent prognosis regarding the recovery of muscle strength [39, 40]. Beneficial immunosuppressive treatment is also reported in the paravertebral manifestation of myositis [41, 42]. In PD, there are only reports of small case series with 1–4 patients receiving immunosuppressive therapy after being diagnosed with (focal) myositis in the paravertebral muscles. In total, 19 patients are reported in 11 case reports, of whom 12 reported improvement as an effect of immunosuppressive therapy [8, 21, 29, 41, 43–49]. EMG and MRI were not specific in these cases, and especially muscle edema in MRI lacked specificity as it has also been reported for other myopathies and denervation [50].

As supposed by Geroin and colleagues [24], management for PD-associated camptocormia, beyond education, includes optimization of anti-PD therapy, physiotherapy, and in some cases, surgery. `Steroids in case of suspected inflammatory PNS condition` is only a minor point in their algorithm, but might be of higher importance if secured by histopathological diagnosis in muscle biopsy. Our results, in addition, do not support the assumption that camptocormia associated with PD might be rather a dystonic dysregulation in muscles of the spine, which would be addressed by botulinum toxin injections.

The strength of this study is the standardized diagnostic investigation of paraspinal muscle tissue in 66 patients with PD-associated camptocormia for the detection of inflammatory findings. The findings of the therapeutic aspect of the study, however, should be considered in the context of several limitations. First, there were no specific referral criteria, but given that all patients had been selected in a tertiary referral center, an overrepresentation of more severe or atypical cases seems possible. Mainly, the investigation is not a randomized clinical trial using objective criteria for endpoint measurement under controlled conditions, but consists of a series of cases with the diagnosis of focal myositis who were treated according to general standards of myositis treatment, primarily irrespective of the paravertebral localization and the associated PD condition. The judgement on clinical change after therapy was not standardized with quantitative measures, but was performed based on global impression of the clinical improvement and the patient´s statement on reduction of clinical burden (without standardized quality of life measures). It has to be noted that the interpretation of the therapy data is limited by the uncontrolled design, the subjective outcome assessment, and limited follow-up. Further studies in a prospective design with standardization of outcome parameters and larger patient samples are thus needed.

In summary, histopathological assessment of muscle biopsies in this cohort of PD patients with camptocormia revealed myopathic changes and in addition local myositis in 64% of biopsies, which then clinically responded to immunosuppressive therapy in two thirds of the treated PD patients. PD-associated camptocormia therefore seems to represent a diagnosis with specific therapeutic options and myositis seems to be a potentially modifiable mechanism in this disabling PD complication. We strongly recommend a diagnostic pathway with histological confirmation by paravertebral muscle biopsy in PD and camptocormia.

## Data Availability

The dataset used (fully anonymized) and analyzed during the current study will be made available by the corresponding author upon reasonable request by qualified researchers.
